# Kaempferol, a Phytoprogestin, Induces a Subset of Progesterone-Regulated Genes in the Uterus

**DOI:** 10.3390/nu15061407

**Published:** 2023-03-15

**Authors:** Tova M. Bergsten, Kailiang Li, Daniel D. Lantvit, Brian T. Murphy, Joanna E. Burdette

**Affiliations:** Department of Pharmaceutical Sciences, College of Pharmacy, University of Illinois Chicago, Chicago, IL 60607, USA

**Keywords:** progestin, phytoprogestin, natural product, kaempferol, apigenin, progesterone

## Abstract

Progesterone functions as a steroid hormone involved in female reproductive physiology. While some reproductive disorders manifest with symptoms that can be treated by progesterone or synthetic progestins, recent data suggest that women also seek botanical supplements to alleviate these symptoms. However, botanical supplements are not regulated by the U.S. Food and Drug Administration and therefore it is important to characterize and quantify the inherent active compounds and biological targets of supplements within cellular and animal systems. In this study, we analyzed the effect of two natural products, the flavonoids, apigenin and kaempferol, to determine their relationship to progesterone treatment in vivo. According to immunohistochemical analysis of uterine tissue, kaempferol and apigenin have some progestogenic activity, but do not act in exactly the same manner as progesterone. More specifically, kaempferol treatment did not induce HAND2, did not change proliferation, and induced ZBTB16 expression. Additionally, while apigenin treatment did not appear to dramatically affect transcripts, kaempferol treatment altered some transcripts (44%) in a similar manner to progesterone treatment but had some unique effects as well. Kaempferol regulated primarily unfolded protein response, androgen response, and interferon-related transcripts in a similar manner to progesterone. However, the effects of progesterone were more significant in regulating thousands of transcripts making kaempferol a selective modifier of signaling in the mouse uterus. In summary, the phytoprogestins, apigenin and kaempferol, have progestogenic activity in vivo but also act uniquely.

## 1. Introduction

Progesterone is a steroid hormone that plays numerous roles in normal human physiology, particularly in the female reproductive system. Progesterone carries out most of these functions by binding to the nuclear progesterone receptors (PRA and PRB) and acting as a transcription factor to regulate downstream target genes [1]. Perhaps most prominently studied are the roles of progesterone in female reproductive tissues including the breasts, ovaries, and uterus [2]. In particular, progesterone is well-known for its ability to counteract estrogen-induced epithelial proliferation in the endometrium [3]. As such, progesterone is prescribed to counteract unwanted symptoms such as premenopausal abnormal bleeding or excessive uterine proliferation in conditions such as endometriosis [4]. These prescriptions are typically for synthetic progestins that bind the progesterone receptor, since progesterone itself is not orally bioavailable unless formulated as micronized progesterone. However, recent data suggest that women have been increasingly turning to botanical supplements to alleviate these and other symptoms, including those from premenstrual syndrome, menopause, and infertility [5,6]. In 2020, consumer spending on herbal supplements increased by over 17% from 2019, reaching over USD 11 billion dollars nationwide [7].

Unfortunately, while these supplements may provide women with alternative treatment strategies, they are often not regulated by the U.S. Food and Drug Administration and therefore have not undergone rigorous testing to identify active compounds, effective doses, inherent toxicity, or drug–supplement interactions. Hence, it is important to characterize and quantify active compounds in botanical supplements, and also their biological targets within cellular and animal systems. Several studies have identified that botanical supplements contain compounds that are capable of binding human steroid receptors and activating their downstream effects. Traditionally, these compounds have included phytoestrogens, which can bind the estrogen receptor, but have more recently been shown to include phytoprogestins as well, which can bind the progesterone receptor [8,9]. 

Two such compounds, the flavonoids apigenin and kaempferol, are found in a variety of fruits, vegetables, and botanicals [10,11]. In vitro, kaempferol demonstrates antioxidant and anti-inflammatory properties as well as anti-proliferative properties in ovarian cancer cells, which is noteworthy as progestins also have an immunosuppressive and anti-inflammatory action [12,13,14]. We previously reported that apigenin and kaempferol were orally bioavailable in an ovariectomized rat model and had progestin-like effects on uterine tissue when administered by oral gavage [15,16]. However, progesterone was unable to be utilized as a control treatment in these studies as it is not orally bioavailable. Therefore, the need to directly compare the effects of apigenin and kaempferol to progesterone remained. To accomplish this, we employed a mouse model where treatments would be provided via intraperitoneal injections allowing for the inclusion of progesterone alongside apigenin and kaempferol, and the subsequent identification and analysis of genome-wide target genes. 

## 2. Materials and Methods

### 2.1. Animal Study and Chemicals

This study utilized ovariectomized 6–8-week-old CD1 mice (Envigo, Indiannapolis, IN, USA). Animals were housed in a temperature- and light (12L:12D)-controlled environment. Water and food were provided ad libitum. Mice were fed with AIN-76A diet (Envigo CA.170481), devoid of phytoestrogens. All animals were treated in accordance with the National Institutes of Health Guide for the Care and Use of Laboratory Animals. Mice were ovariectomized by the supplier and treatment began no sooner than two weeks after surgery to ensure sufficient time to minimize endogenous hormone effects. 

Progesterone (≥99% purity, Sigma-Aldrich, St Louis, MO, USA, P0130) was used at 1 mg/kg. Kaempferol (≥98% purity, Cayman Chemical, Ann Arbor, MI, USA, 11852) and apigenin (≥98% purity, Cayman Chemical 10010275) were each used at 5.625 mg/kg. These doses were utilized to maintain consistency with our previous studies which utilized 5.625 mg/kg of either compound [15,16]. Five mice were randomly assigned into each treatment group and received once a day intraperitoneal injection of either drug dissolved in 10% DMSO for 7 days. 

At day 7, mice were weighed and euthanized via humane means (asphyxiation via CO_2_ and cervical dislocation) prior to the collection and weight of their uterine tissue. One uterine horn was snap-frozen in liquid nitrogen and stored at −80 °C for RNA extraction. The second uterine horn was fixed in 10 mL of 10% buffered formalin for 24 h, transferred into 70% EtOH, and processed for histology using a Shandon 1000 processor (Thermo, Waltham, MA, USA). Processed tissue was then embedded with paraffin into 5 mm thick blocks and sectioned into 5 µm sections with a microtome. This study was approved by the UIC Institutional Animal Care and Use Committee (protocol number 18-205).

### 2.2. Immunohistochemical Staining

IHC was performed for proliferating cell nuclear antigen (PCNA), zinc finger and BTB domain-containing 16 (ZBTB16), heart and neural crest derivates-expressed protein 2 (HAND2), and FK506 binding protein 5 (FKBP5) on uterine samples as previously described [17]. The tissue sections were incubated with the following primary antibodies overnight at 4 °C: PCNA (1:200, 13,110 Cell Signaling, Danvers, MA, USA), HAND2 (1:200, ab200040 Abcam, Cambridge, UK), FKBP5 (1:200, 14155-1 Protein Tech, Rosemont, IL, USA), and ZBTB16 (1:200, PA5-112862 Invitrogen, Waltham, MA, USA). RRIDs for these antibodies are as follows: PCNA (AB_2636979), HAND2 (AB_2923502), FKBP5 (AB_2231625), and ZBTB16 (AB_2867596). Subsequently, slides were incubated with anti-goat biotinylated secondary antibody (Vectastain ABC kit; Vector Laboratories, Inc., Burlingame, CA, USA) at 1:200 dilution in PBST for 60 min at room temperature. Slides were imaged using a Nikon E600 Eclipse microscope with a CMOS C-Mount microscope camera.

PCNA expression was counted for each stained cell in either glandular or luminal epithelium. The number of stained cells was graphed using GraphPad and a one-way ANOVA was used to determine significance (* *p* < 0.05). Expression of ZBTB16, FKBP5, and HAND2 in treatment-blinded representative images was determined on a scale of 0 to +3 by distinct viewers. These results were graphed using GraphPad and a one-way ANOVA was used to determine significance (* *p* < 0.05). 

### 2.3. RNA Isolation and RNA Sequencing Profiling

Uteri of mice treated with 10% DMSO, 1 mg/kg progesterone, or 5.625 mg/kg kaempferol or apigenin for 7 days in the first animal study were subjected to RNA isolation and RNA sequencing. RNA sequencing of uterine tissue was profiled (n = 4 per treatment group). Total RNA was extracted from uterine tissues of mice using the Qiagen RNeasy mini kit (Qiagen, Hilden, Germany, #74104) according to the manufacturer’s instructions. The concentration of mRNA was determined by a Nanodrop. RNA libraries (three technical replicates/treatment) were created. The Genomics Core Facility at Northwestern University performed RNA quality determination, mRNA enrichment, library construction, sequencing, and transcriptome statistical analysis. Samples with RINs of 7 or greater were prepared with TruSeq mRNA-Seq Library Prep (Illumina, San Diego, CA, USA) with 1 μg of RNA and 12 cycles of PCR amplification. The libraries were barcoded, pooled, and sequenced on the HiSeq Sequencing 50 followed by statistical analysis. 

### 2.4. Statistical and Bioinformatics Analysis

For RNAseq data, gene set enrichment of differentially expressed genes was performed using GSEA. Gene sets with an FDR adjusted *p*-value of <0.05 were considered significant. 

## 3. Results

### 3.1. Kaempferol and Apigenin Have Progestogenic Activity In Vivo

In this study, CD-1 mice (*n* = 5/group) were injected intraperitoneally (IP) with 10% DMSO, 1 mg/kg progesterone (P4), 5.625 mg/kg kaempferol, or 5.625 mg/kg apigenin once a day for 7 days. To evaluate the progestin-like effects of each treatment, mouse uteri were collected and subjected to immunohistochemistry (IHC) to determine the protein levels of progesterone-receptor-regulated genes such as HAND2, ZBTB16, FKBP5, and PCNA. 

**Figure 1:**
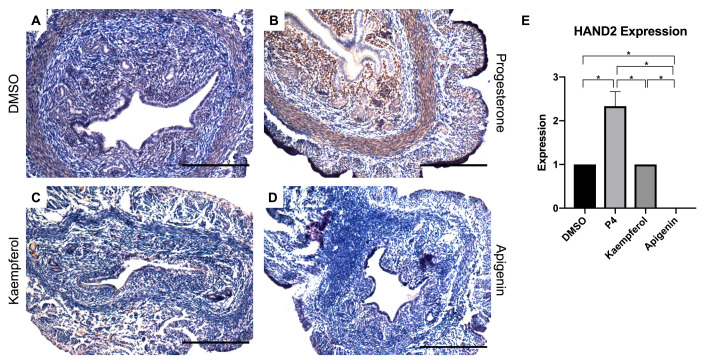
Kaempferol and apigenin did not increase HAND2 expression in mouse uteri. Immunohistochemical staining against progesterone receptor target HAND2 on uterine cross sections of control- (**A**), progesterone- (**B**), kaempferol- (**C**,**D**) apigenin-treated mice. Scale bar = 100 µm; magnification 20×. (**E**) Quantification of HAND2 expression per treatment in representative images. One-way ANOVA test was used to determine significance (* *p* < 0.05).

Heart and neural crest derivates-expressed protein 2 (HAND2) is known to regulate a specific function in uterine epithelium and is upregulated by P4 in ovariectomized mice [18,19]. Specifically, HAND2 expression in the stromal cells of the uterus is required for secretion of hedgehog signaling factors that then block estrogen-induced proliferation in uterine epithelial cells through paracrine signaling. Compared to the control (Figure 1A), progesterone-treated mice expressed significantly increased HAND2 protein in the stromal cell compartment (Figure 1B,E). While HAND2 expression in kaempferol-treated mouse uteri was more similar to DMSO-treated tissue than progesterone-treated tissue (Figure 1C,E), apigenin treatment seemed to have no effect on HAND2 expression (Figure 1D,E). This result in mice differs from our previous work in which compounds were given orally to rats and both apigenin and kaempferol increased HAND2 expression in uterine tissues [15,16]. 

**Figure 2:**
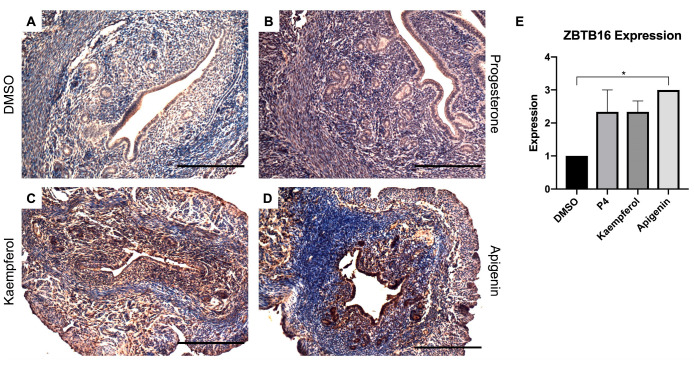
Kaempferol and apigenin increased ZBTB16 expression in mouse uteri. Immunohistochemical staining against progesterone receptor target ZBTB16 on uterine cross sections of control- (**A**), progesterone- (**B**), kaempferol- (**C**,**D**) apigenin-treated mice. Scale bar = 100 µm; magnification 20×. (**E**) Quantification of ZBTB16 expression per treatment in representative images. One-way ANOVA test was used to determine significance (* *p* < 0.05).

**Figure 3:**
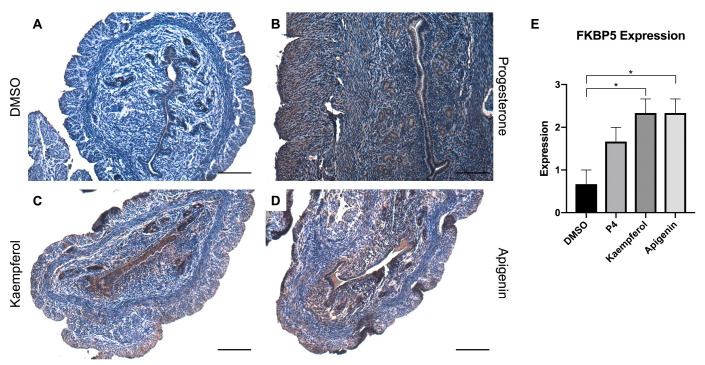
Kaempferol and apigenin increased FKBP5 expression in mouse uteri. Immunohistochemical staining against progesterone receptor target FKBP5 on uterine cross sections of control- (**A**), progesterone- (**B**), kaempferol- (**C**,**D**) apigenin-treated mice. Scale bar = 100 µm; magnification 20×. (**E**) Quantification of FKBP5 expression per treatment in representative images. One-way ANOVA test was used to determine significance (* *p* < 0.05).

Zinc finger and BTB domain-containing 16 (ZBTB16) is also induced by P4 and known to play a role in stromal cell decidualization [20]. In this study, progesterone (Figure 2B) and kaempferol (Figure 2C) treatments seemed to slightly increase ZBTB16 expression when compared with DSMO (Figure 2A), although these changes were not statistically significant (Figure 2E). However, apigenin (Figure 2D) significantly increased ZBTB16 protein levels as compared to DMSO as seen in IHC (Figure 2A,E). FK506 binding protein 5 (FKBP5) is involved in the assembly of the glucocorticoid receptor complex and functions as an inhibitor of its assembly until it disassociates from the complex [21]. However, the *fkbp5* gene is inducible by glucocorticoids, as well as progesterone and androgenic hormones [22,23,24,25,26,27,28]. While FKBP5 expression was seemingly increased by progesterone (Figure 3B) treatment, only kaempferol (Figure 3C) and apigenin treatments (Figure 3D) induced significantly increased ZBTB16 expression when compared with control (Figure 3A,E). 

**Figure 4:**
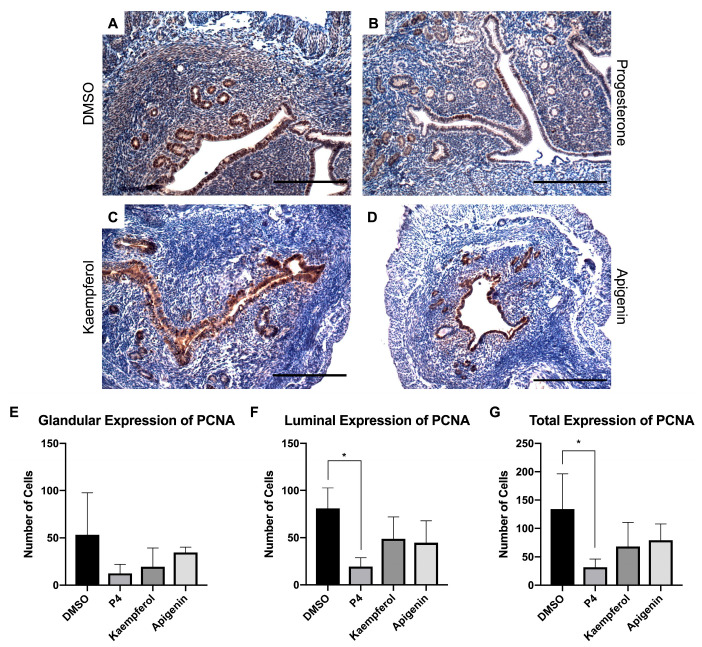
Kaempferol and apigenin did not inhibit luminal and glandular epithelial cell proliferation. Immunohistochemical staining against proliferation marker PCNA on uterine cross sections of control- (**A**), progesterone- (**B**), kaempferol- (**C**,**D**) apigenin-treated mice. Scale bar = 100 µm; magnification 20×. Quantification of the number of PCNA cells per treatment in representative images in the (**E**) glandular, (**F**) luminal, or (**G**) total epithelium. One-way ANOVA test was used to determine significance (* *p* < 0.05).

Additionally, we investigated the effects of these compounds on uterine epithelial proliferation. It is well established that progesterone inhibits estrogen-induced uterine epithelial proliferation through the progesterone receptor [3]. Importantly, the mice in this study were ovariectomized thereby reducing endogenous hormone production. In this model, progesterone-treated mouse uteri displayed a decreased expression of proliferating cell nuclear antigen (PCNA) (Figure 4B), an epithelial proliferation marker, when compared with the DMSO-treated mouse uteri (Figure 4A,E). While kaempferol and apigenin did not appear to significantly decrease PCNA expression in the epithelium, there was seemingly less expression in the stroma than in the control-treated tissue (Figure 4C–G). In a previous study, although apigenin increased expression of Ki67, another proliferation marker, it increased expression to a lesser degree than the phytoestrogen genistein and was able to block the genistein-induced Ki67 increase in combination treatment [15]. Kaempferol had a similar profile in another study, increasing Ki67 expression when compared with control but also counteracting the estrogenic proliferation induced by genistein [16]. These data underscore the role of progesterone receptor-mediated signaling in the presence and absence of estrogen receptor activation and indicate that both apigenin and kaempferol block genistein-induced signaling but do not reduce basal levels of uterine epithelial proliferation.

### 3.2. Kaempferol and Progesterone Effects on mRNA Transcripts 

To investigate and compare the transcriptomic profiles of tissues collected from mice injected with progesterone, kaempferol, and apigenin, we extracted mRNA from the uteri of these mice (*n* = 4/group) and performed next generation RNA sequencing (a full list of altered transcripts is available in Table S1). Apigenin did not appear to regulate more than a handful of transcripts, suggesting poor bioavailability when given intraperitoneally. As such, the majority of our analysis focused on the effects of progesterone and kaempferol treatments. 

**Figure 5:**
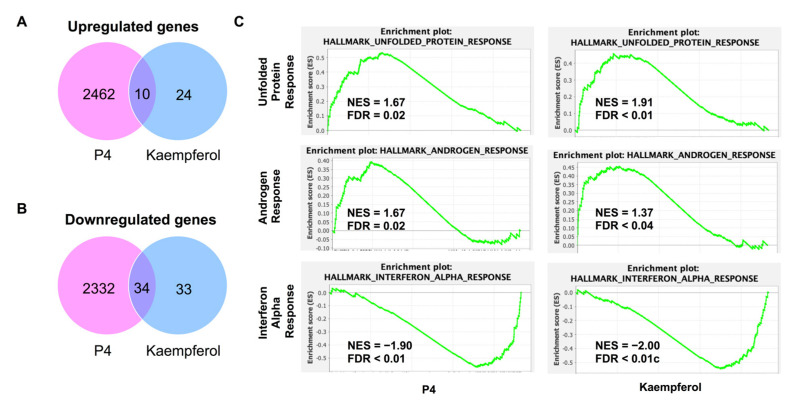
(**A**) Significantly upregulated genes found in RNAseq of P4 and kaempferol-treated uterine tissue. (**B**) Significantly downregulated genes found in RNAseq of P4 and kaempferol-treated uterine tissue. (**C**) Similarly regulated GSEA pathways between P4 and kaempferol treatments.

Progesterone treatment altered significantly more transcripts than were found to be altered in kaempferol-treated tissues. Progesterone upregulated 2472 transcripts while kaempferol upregulated only 34 transcripts; 10 transcripts were upregulated by both treatments (Figure 5A, Table 1). Progesterone downregulated 2366 transcripts while kaempferol downregulated only 67 transcripts; 34 transcripts were downregulated by both treatments (Figure 5B, Table 2). Roughly half (44%) of all the transcripts regulated by kaempferol were also regulated by progesterone treatment. A total of 29% of the upregulated transcripts and 51% of the downregulated transcripts were altered by both kaempferol and progesterone treatments. Lefty1 was the most upregulated transcript (2.61-fold), while Cemip was the most downregulated transcript (−1.43-fold) in kaempferol-treated tissues. 

RNAseq data were then subjected to GSEA analysis to identify significantly altered gene sets [29,30]. Similarly altered pathways were found for both progesterone and kaempferol treatments including unfolded protein response (UPR), androgen response (AR), and interferon alpha response (IAR) (Figure 5C). The normalized enrichment score (NES) values for the UPR and AR pathways were positive for both progesterone- and kaempferol-treated tissues, implying that the majority of altered genes in these pathways were upregulated in the tissues of both treatments. According to GSEA, the transcripts in the UPR pathway, such as ATF3, are typically upregulated in a cellular stress response related to the endoplasmic reticulum (ER). In the corpus luteum, UPR signaling pathway activation has been shown to help maintain progesterone expression during luteal phase progression [31]. Further, ER stress-induced apoptosis mediated by CHOP and the caspase cascade, parts of the UPR pathways, has been shown to be involved in the regression of the corpus luteum [31,32]. These data suggest that UPR pathways play a role in the regulation of the corpus luteum and progesterone production. In prostate cancer cells, progesterone treatment induced several UPR pathway proteins [33]. Additionally, both in vitro treatment with progesterone and uterine tissue analyzed during the secretory phase of the uterine cycle, which is characterized by high levels of progesterone, showed increased CHOP expression and apoptosis in endometrial cells [34]. These data suggest that progesterone is both capable of regulating and being regulated by the UPR pathway.

Unsurprisingly, progesterone treatment upregulated transcripts defining of the androgen response since progesterone signaling is canonically deeply intertwined with androgen signaling. Progestins are known to bind similar DNA response elements as androgens and have been known to act as partial androgen receptor agonists, which is often the suggested rationale for why progestins can cause seemingly androgen-induced side-effects [35,36]. The similar pattern of gene alteration in these pathways seen in both kaempferol- and progesterone-treated tissues is indicative of kaempferol acting as a progestin. In contrast, the NES value for the IAR pathway was negative for both treatments. This suggests that the majority of altered genes in the IAR pathway, such as RSAD2, were downregulated in both sets of tissues, or that both progesterone and kaempferol treatment attenuate the transcription of genes canonically upregulated in response to alpha interferon proteins meant to handle a viral infection.

## 4. Discussion

Synthetic progestins are widely sought as treatments for many unwanted symptoms originating in female reproductive tracts. While these molecules have current practical applications in women’s health, the natural compounds that function to modify PR signaling in botanical dietary supplements remain less well studied. As such, the need to understand the contents of these supplements and their numerous effects is ever growing. For this reason, we undertook an in vivo study to determine the effects of two previously identified phytoprogestins, apigenin and kaempferol, and their similarity to progesterone treatment. 

According to our immunohistochemistry results, kaempferol and apigenin have some progestogenic activity, but do not act in exactly the same manner. For instance, while progesterone treatment upregulated the known progesterone target gene HAND2, neither kaempferol nor apigenin upregulated HAND2 expression to the same extent. However, both kaempferol and apigenin increased ZBTB16 and FKBP5 expression, other progesterone inducible genes, as did progesterone. Interestingly, FKBP5 is one of the main drivers of the androgen response pathway in GSEA that was upregulated by progesterone in our RNAseq data. While kaempferol treatment did not significantly upregulate FKBP5 expression according to RNAseq, this may be due to a single replicate of RNA as the IHC suggests kaempferol does increase FKBP5 expression at the protein level. Both kaempferol- and apigenin-treated tissues stained for PCNA demonstrated higher expression of PCNA in the luminal epithelium than in those of tissues treated with progesterone. This aligns with previous data suggesting that while kaempferol is not able to suppress PCNA expression alone, it is able to decrease proliferation when given in combination with an estrogenic compound such as genistein [16]. These data suggest that both kaempferol and apigenin have some progestogenic activity. However, there remains more to learn about the extent of their activity in vivo, particularly comparing mouse and rat uteri. 

RNAseq data determined that progesterone treatment altered many more transcripts than kaempferol treatment. However, while kaempferol treatment did alter some transcripts in a similar manner to progesterone treatment, kaempferol treatment also had some unique effects. A few of the transcripts upregulated only by kaempferol treatment, including LEFTY1 and MMP3, are known to be altered during menstruation. For instance, MMP3 has been shown to increase in ovarian granulosa cells exposed to P4 treatment, suggesting that its upregulation in our data could be evidence of kaempferol’s progestogenic action [37]. Our study also showed an upregulation of LEFTY1 transcripts due to kaempferol treatment. Interestingly, LEFTY1 is expressed all throughout the estrus cycle but has been shown to induce MMP3 upregulation, which may suggest a relationship between these transcripts and their regulation by progesterone [38,39]. 

Several transcripts upregulated by both progesterone and kaempferol treatments are known to play a role in successful implantation of a conceptus into appropriately decidualized endometrium, a process regulated by PR signaling. In a human model of implantation, ATF3 was shown to promote adhesion of spheroids to endometrial cells and has been shown to be decreased in the endometria of patients experiencing recurrent implantation failure [40]. Additionally, knockdown of ATF3 in a human endometrial model impaired decidualization, suggesting a rationale for the recurrent implantation failure seen in patients with insufficient amounts of ATF3 [41]. ATF3 is also a hallmark gene in the UPR pathway that was identified as having several transcripts significantly upregulated by both kaempferol and progesterone treatments. Similarly, SFRP4 has been shown to be highly expressed in decidualizing endometrium [42]. SFRP4 has also been identified as significantly downregulated in uterine lavage samples of infertile women, and this downregulation may affect the proper endometrial development necessary for successful implantation [43]. Lrp2 expression, which was upregulated by kaempferol 2.61-fold over progesterone in the RNAseq data, is known to be upregulated by progesterone treatment and reach its peak at the implantation window in mice endometria [44]. These data suggest that kaempferol treatment may have progestogenic properties in that it similarly upregulates many transcripts involved in appropriate endometrial preparations for successful implantation. In contrast, MRAP2, upregulated by kaempferol and progesterone treatments, has been identified as downregulated in human pre-receptive and receptive endometria and upregulated in the endometria of infertile patients [45,46]. These data suggest that both progesterone and kaempferol may not have completely isolated pro-implantation affects. Future studies could focus on kaempferol treatment and its regulation of decidualization and implantation.

Interestingly, many more transcripts were downregulated by kaempferol treatment than were upregulated. This is congruent with the literature demonstrating that when steroid hormones, such as estrogen, bind to nuclear receptors, the majority of altered transcripts are repressed rather than activated [47,48]. Many of the transcripts downregulated by kaempferol are involved in fertility or the implantation process of pregnancy. For example, knockout of 4930447C04Rik or Six6ox1, which was only downregulated by kaempferol, in female mice results in oocyte insufficiency causing infertility [49]. In mice with knockout of Asb4−/−, here downregulated by both progesterone and kaempferol treatments, there were many placental development issues resulting in decreased fertility [50]. Similarly, Nr5a2, downregulated by kaempferol only, is required for healthy placental formation and the promotion of decidualization in both mouse and human models [51,52]. While these data suggest that kaempferol treatment may repress the expression of genes required for successful pregnancies, we saw that kaempferol treatment activates expression of transcripts necessary for healthy pregnancies as well. Therefore, more research is needed to fully understand the relationship between kaempferol treatment and implantation events.

Other transcripts that were downregulated by kaempferol treatment are shown in the previous literature to be related to cancer. These genes are of interest due to the association in the literature between progesterone-only contraceptive use and reduced risk of ovarian and endometrial cancer. CEMIP, downregulated by both kaempferol and progesterone treatments, is upregulated in the tumor tissues of patients with epithelial ovarian cancer [53]. Knockdown of CEMIP is also shown to decrease oncogenic properties, including proliferation, invasion, and migration, in a human ovarian cancer cell line [53]. Additionally, OGDHL is upregulated in epithelial ovarian cancer tumor samples compared with controls but downregulated by both kaempferol and progesterone treatments [54]. Similarly, both kaempferol and progesterone treatment downregulated RSAD2 which is both upregulated and associated with poorer progression in patients with endometrial adenocarcinoma [55]. RSAD2 is also a hallmark gene of the IAR pathway, transcripts of which were significantly downregulated by both progesterone and kaempferol treatment. These results suggest that kaempferol treatment may decrease expression of transcripts involved in these specific cancer types. However, OSR2 expression, decreased only by kaempferol treatment, is commonly downregulated in endometrial cancer suggesting that some transcripts associated with risk have varied expression patterns as a result of kaempferol treatment [56]. 

Another trend our RNAseq data identified was that several genes downregulated by kaempferol treatment are related to polycystic ovary syndrome (PCOS) according to the literature. PCOS is characterized by the formation of many cysts on the ovaries, which also produce an abnormally high level of androgens and low circulating levels of progesterone due to reduced ovulation. Expression of SLC5A3, decreased by kaempferol treatment, was also found to be decreased in the endometrial tissue from women with PCOS [57]. Further, this decreased expression contributed to the development of insulin-resistance commonly seen in PCOS patients. Similarly, decreased expression of Procr, which was also decreased by both progesterone and kaempferol treatment, led to disrupted ovarian follicle development and a PCOS phenotype in mice [58]. CXCL14 transcripts, decreased by both progesterone and kaempferol treatment, have been shown to be decreased in human luteinized granulosa cells from women with PCOS [59]. Additionally, a lack of CXCL14 in these cells likely contributes to their decreased ability to produce progesterone as treatment with increasing doses of CXCL14 consistently increased progesterone production [59]. Pdgfd expression was also decreased by kaempferol treatment. In women with PCOS, both follicular fluid and serum were found to contain decreased amounts of PDGFD protein when compared with samples from control patients [60,61]. Further, PDGFD levels were found to be decreased in a rat model of PCOS [62]. These data suggest that kaempferol treatment may be inducing a similar gene expression as those seen in PCOS models, as supported by one of the significant GSEA pathways being related to androgen signaling. However, kaempferol and progesterone treatment both decreased C3 transcript expression, which is shown to be upregulated along with other complementary pathway factors in patients with PCOS and is thought to contribute to the inflammatory aspect of the disease [63,64]. This suggests that the effects of kaempferol treatment on PCOS pathways and regulated transcripts may not be entirely consistent and require more investigation.

In summary, these data suggest that both apigenin and kaempferol have progestogenic activity in vivo in a murine model. For instance, immunohistochemical analysis shows that both apigenin and kaempferol increase ZBTB16 and FKBP5 expression in the uterus similarly to progesterone. RNA sequencing data further support that kaempferol has progestogenic activity, although in general regulated far fewer genes than progesterone, due to the similar modulation of transcripts when compared with progesterone treatment. While these transcripts have some relationship with reproductive health, future studies are required in order to more fully understand the effects of kaempferol treatment. For instance, while we anticipate that kaempferol will maintain progestogenic activity in gonadally intact female mice, this would be a greatly beneficial next step in the investigation into the role of kaempferol in vivo.

## Figures and Tables

**Table 1:** Transcripts significantly upregulated by kaempferol treatment when compared with vehicle-treated tissue.

Gene Symbol	Kaempferol Log_2_ Fold Change	Kaempferol *p* Value	Upregulated by P4
Lefty1	2.61	4.83 × 10^−21^	No
Padi4	2.08	3.06 × 10^−17^	Yes
Sdr9c7	1.89	8.53 × 10^−12^	No
Gm5148	1.77	1.41 × 10^−10^	No
Muc5b	1.71	8.04 × 10^−10^	No
Syt8	1.41	4.27 × 10^−7^	No
Mmp3	1.38	5.92 × 10^−7^	No
Msr1	1.34	1.35 × 10^−6^	No
Ceacam19	1.29	1.68 × 10^−6^	No
Atf3	1.27	1.61 × 10^−6^	Yes
Fam83c	1.25	6.64 × 10^−6^	No
Mrap2	1.22	5.30 × 10^−6^	Yes
Prss27	1.21	7.34 × 10^−6^	No
Sfrp4	1.21	1.19 × 10^−7^	Yes
Galnt6	1.15	3.31 × 10^−5^	No
Hk3	1.12	4.21 × 10^−5^	No
Rarb	1.12	3.36 × 10^−5^	No
Pstpip2	1.07	1.12 × 10^−4^	No
Sulf2	1.05	8.77 × 10^−6^	Yes
Fabp5	1.05	1.62 × 10^−4^	No
Brinp3	1.05	5.10 × 10^−5^	No
Atp6v0d2	1.03	1.80 × 10^−4^	No
Lrp2	0.99	4.56 × 10^−5^	Yes
Slamf8	0.96	2.20 × 10^−4^	No
Car12	0.94	4.12 × 10^−6^	No
Padi3	0.93	2.29 × 10^−4^	No
Noct	0.88	7.37 × 10^−5^	Yes
AI467606	0.85	1.73 × 10^−5^	No
Ptgr1	0.82	7.65 × 10^−5^	No
Rhbdl2	0.81	2.88 × 10^−4^	No
Palm3	0.80	3.00 × 10^−4^	Yes
Kdm8	0.74	2.60 × 10^−4^	Yes
Ada	0.74	2.20 × 10^−4^	No
Rab27a	0.72	9.16 × 10^−5^	Yes

**Table 2:** Transcripts significantly downregulated by kaempferol treatment when compared with vehicle-treated tissue.

Gene Symbol	Kaempferol Log^2^ Fold Change	Kaempferol *p* Value	Downregulated by P4
Cemip	−1.43	6.93 × 10^−14^	Yes
Tcte2	−1.42	5.16 × 10^−8^	Yes
2700054A10Rik	−1.42	5.16 × 10^−8^	Yes
Egfl6	−1.29	4.04 × 10^−9^	Yes
C1s1	−1.27	9.21 × 10^−9^	No
Wif1	−1.27	1.96 × 10^−6^	Yes
Lepr	−1.26	1.87 × 10^−7^	No
Bpifb5	−1.25	9.14 × 10^−8^	Yes
A330023F24Rik	−1.22	1.10 × 10^−5^	No
Wnt9b	−1.20	6.24 × 10^−6^	Yes
4930447C04Rik	−1.14	2.24 × 10^−5^	No
Lrrtm1	−1.12	2.45 × 10^−5^	Yes
Alox12e	−1.11	6.04 × 10^−6^	No
Gm525	−1.11	6.75 × 10^−5^	No
Nkain4	−1.04	1.20 × 10^−4^	No
Rnase1	−1.03	2.11 × 10^−4^	Yes
Kcne2	−1.03	2.15 × 10^−4^	Yes
Rsad2	−1.03	8.44 × 10^−5^	Yes
Erich3	−1.00	2.76 × 10^−4^	Yes
Slc5a3	−0.99	7.65 × 10^−6^	No
Car4	−0.99	1.39 × 10^−4^	Yes
Cyp4b1	−0.98	2.00 × 10^−5^	No
C1s2	−0.98	6.63 × 10^−6^	No
Edil3	−0.97	7.49 × 10^−6^	Yes
Mycn	−0.97	1.23 × 10^−4^	Yes
Wipf3	−0.96	4.71 × 10^−5^	No
Asb4	−0.95	2.25 × 10^−10^	Yes
Kctd4	−0.93	2.05 × 10^−5^	No
Ubc	−0.92	4.22 × 10^−6^	No
Procr	−0.91	1.36 × 10^−6^	Yes
C3	−0.91	1.59 × 10^−4^	Yes
Abcb1a	−0.87	9.87 × 10^−5^	No
Selp	−0.86	2.79 × 10^−4^	No
Creb5	−0.85	2.07 × 10^−4^	No
Enkur	−0.85	3.66 × 10^−6^	Yes
Grb14	−0.85	1.47 × 10^−5^	Yes
Gmpr	−0.85	2.77 × 10^−4^	Yes
Nr5a2	−0.83	9.12 × 10^−5^	No
Car8	−0.83	2.85 × 10^−5^	Yes
Osr2	−0.77	4.65 × 10^−8^	No
Ogdhl	−0.77	9.33 × 10^−7^	Yes
Cyp46a1	−0.75	2.33 × 10^−4^	No
Cxcl14	−0.74	5.47 × 10^−5^	Yes
2700046A07Rik	−0.72	2.10 × 10^−4^	Yes
Nrep	−0.71	5.24 × 10^−6^	Yes
Usp2	−0.69	2.17 × 10^−4^	No
Prdm1	−0.69	4.33 × 10^−5^	Yes
Pdgfd	−0.69	2.62 × 10^−4^	No
3632451O06Rik	−0.68	2.72 × 10^−4^	No
C130074G19Rik	−0.67	1.68 × 10^−4^	Yes
Zhx3	−0.66	1.29 × 10^−4^	No
Csrp2	−0.63	9.08 × 10^−6^	No
Afap1l1	−0.62	2.67 × 10^−9^	Yes
Rapgef4	−0.61	1.42 × 10^−4^	No
Pfkfb3	−0.61	2.89 × 10^−5^	No
Grrp1	−0.59	9.37 × 10^−6^	Yes
Gimap6	−0.59	1.31 × 10^−4^	No
Kank3	−0.58	1.31 × 10^−6^	No
Ly6c1	−0.56	1.40 × 10^−6^	Yes
Sox18	−0.55	1.16 × 10^−4^	No
Esam	−0.53	1.30 × 10^−5^	Yes
Pdgfc	−0.52	8.70 × 10^−5^	Yes
Zfp503	−0.51	2.87 × 10^−4^	No
Casp4	−0.51	4.23 × 10^−5^	Yes
Palmd	−0.50	1.84 × 10^−4^	No
Cdc42ep1	−0.47	1.35 × 10^−4^	No
Ushbp1	−0.43	1.26 × 10^−4^	No

## Data Availability

Not applicable.

## Supplementary Materials

The following supporting information can be downloaded at: https://www.mdpi.com/article/10.3390/nu15061407/s1, Table S1: RNAseq Altered Transcripts.
